# Annals of Quackery—II

**Published:** 1910-06-18

**Authors:** 


### SPECIAL ARTICLES.

### ANNALS OF QUACKERY—II.

To proceed with the story of the extraordinary campaign against quackery which was undertaken in the early part of last century, it should be observed that the quacks against whom the attacks were levelled were more especially those who posed as physicians and surgeons in addition to providing their victims with "cures." Those who merely sold advertised nostrums were treated with less severity; in fact, it is explained in one of the articles that some of the commonly-sold nostrums were really beneficial, as witnessed by the fact that the formulæ for several were subsequently introduced into the Pharmacopœia. On the other hand, it is complained that "the druggists have lately got into the habit of not only taking upon themselves the duties of professionally educated men, but each makes up some pill of his own, which he sells enormously dear." These are described as productions of ignorance and stupidity which bring on "an habitual state of costiveness." They, however, were not made the subject of such special condemnation as the quack doctors. Of the latter something like one hundred are exposed in different issues of the Journal, and it will be interesting to select a few of these in order to show both the kind of men they were and the fearless manner in which they were attacked. In the first place there was "Dr." Twynham, the bone-setter and "general doctor" of Kingsland Road. When his time for exposure came he had already been practising his inspired knowledge for upwards of forty years—that is to say since 1780. This man, according to his business card, cured the most dreadful disorders, and ended a pompous enumeration by "N.B.—Warts cured." In a comment on his methods, it is said, "were the list of Twynham's patients alone to be published, with their cases, mode of treatment, and their results, a monument of English credulity and stupidity would be transmitted to posterity, which even our libraries of philosophy could not redeem."

A quack of a somewhat different order was "Dr." Gardner, who appears to have specialised in the treatment of worms. The description of this man's "vermicular depôts" in Holywell Street, Shoreditch, and Long Acre, is indeed vivid; according to the account, in his shop were set out to the best advantage "a vast number of bottles of various dimensions, containing worms of every species, natural and artificial, from the dimensions of an atom to several hundred yards in length." All these were labelled with details of whence they came—always by the effect of Gardner's medicine. In the parlour, behind the shop, sat a prim-looking, smoothed out, plaited-capped old woman, watching through the glass the gazers outside the window, and reading the effects of the labels upon their simple countenances. As soon as a customer came in she slowly rose, heard with a grave aspect the application, and then went through her threadbare lesson in the following purport:—

"I see, sir, you want the doctor!"

"Why, ma'am, I want something to do me good."

"Ha, yes, yes, yes, I see you do, but the doctor is now at chapel, offering up thanks to God for the recovery of a lady that was on the brink of the grave. I see, young man, you suffer from worms."

"Very likely, ma'am."

"Yes, yes, yes, here is a sort of reptile that is killing you"; and then, taking down a bottle, wipes it clean, and shows the contents to the terrified patient.

"This, sir, is the horrid viper that preys upon your entrails, and the person that this *effected* was cured with a thirty-shilling box of the 'Doctor's' pills, which we can now sell for a guinea, thanks be to God! There, sir, look at its fourteen pairs of horns and its six-and-thirty mouths. It is called by physicians the wormus magnus."

The poor victim is frightened; he instantly demands whether the pills will do him good, and is assured that a guinea box will restore him to health.

Concerning Gardner *The Medical Adviser* says: "The most arrant pickpocket that ever was transported to Botany Bay, who, while exhorting, plundered you, could with as much propriety take up the practice of religion as this old worm dealer." Of his display of worms it is said that they were collected from different sources, either bought or composed, as a showman does his menagerie and stuffed animals.

Another quack singled out for attack is "Dr." Mitchell, who frequented 'Change at the hour of bustle. He is minutely described as "a thin sallow-looking fellow, about forty-eight, five feet seven inches in height, with powdered hair, a silky sort of hat, and a jalapy suit of black. His countenance possesses a mixture of impudence and embarrassment rising out of his wish to catch *new* customers, and to avoid the *old*. Like his brethren the pickpockets, his eyes are eternally divided in their duty—keeping out of his *done* customers' way and full butting the "customers to do." "Dr." Mitchell started life as a work-boy to an old-clothes man in Rosemary Lane, then gave out bills to the passengers on Tower Hill for an old quack, and ultimately set up for himself and selected his patients chiefly among seafaring men.

Another irregular practitioner was "Dr." Courtney, whose address is given at "Adam Street, Adelphi, and the Rules of the Fleet!" He is described as one of the most dangerous men in the country—"the midnight wolf, loose amidst the unsuspecting flock, is not capable of the ravages that follow from this audacious presumption and roguery. He is the most dangerous of all the other quacks because, from his being such an adept in swindling, he has put on, in all its exterior dignity, the stolen robe of the regular and educated practitioner." His specialty was the treatment of stricture, on which he produced a book of which several pages contained "a few of the heterogeneous remarks of various medicinal writers, ignorantly and obscurely thrown together in disguised language." The rest of the book was filled up with cases evidently constructed to meet a similarity in those who chanced to read it. Case the first is the Honourable A. G., the second a colonel in the army, the third Captain R. of the Royal Navy, the fourth Captain P. of the Royal Marines, the fifth O. B., Esq., from Ireland, the sixth a proprietor of coaches, the seventh T. R., a merchant, etc., etc. In fact, the nobility, gentry, army, navy, merchants, and traders took their places in "Dr." Courtney's list according to their respective ranks. As to fees it is stated that thirty guineas is a common sum "for him to trick a man out of." Perhaps, not unnaturally, "Dr." Courtney took exception to the remarks concerning him, and the statement in a subsequent issue that "Courtney sent a tall, lawyer-looking gentleman to our publishers with a polite letter in one hand and a threat in the other," need occasion no surprise.

